# A Rare Case of Congenital Knee Dislocation

**DOI:** 10.7759/cureus.88456

**Published:** 2025-07-21

**Authors:** Mohammed M Mumtaz, Molly R Posa, Elahe M Shychuk

**Affiliations:** 1 Pediatrics, University of Florida College of Medicine, Gainesville, USA

**Keywords:** congenital dislocation of the knee, congenital hip dislocation, congenital orthopedic abnormalities, developmental dysplasia of the hip, neonatal orthopedic abnormalities, packaging disorders, pavlik harness

## Abstract

Congenital knee dislocation (CKD) is a rare condition that can present as an isolated finding or in association with genetic syndromes. It is most commonly diagnosed postnatally and classified into three grades based on severity. While generally having a favorable prognosis, particularly for milder cases, prompt treatment is crucial. We present a case of CKD successfully treated with conservative measures, with serial casting and Pavlik harness, in an otherwise healthy infant. This report aims to increase awareness of the clinical presentation and management of CKD in general practitioners given its rarity.

## Introduction

Congenital knee dislocation (CKD) is a rare condition that involves dislocation of the knee joint in utero, often accompanied by hyperextension of the knee. It can be an isolated idiopathic finding, in association with genetic syndromes, or can be a consequence of intrauterine compression deformities [1]. It is estimated to occur in approximately one in 100,000 live births [2]. While CKD has been identified prenatally, it is most commonly diagnosed after delivery, characterized by hyperextension of the knee with anterior displacement of the proximal tibia [3]. CKD can be classified into the following three types: simple hyperextension (Grade 1), anterior tibial subluxation reducible with flexion (Grade 2), and anterior tibial dislocation (Grade 3) [4]. The prognosis for CKD is generally favorable, especially in Grade 1 and Grade 2 cases. First-line treatment typically involves a combination of closed reduction and serial casting, while more severe forms may require surgical intervention [5]. Although less commonly documented, earlier literature spanning more than four decades suggests that the Pavlik harness, normally a treatment for developmental dysplasia of the hip (DDH), can have a therapeutic effect for newborns with mild isolated CKD [6]. Given the rarity of CKD, it may be unfamiliar to many general pediatricians. This report aims to increase awareness of its clinical presentation. Early recognition is important, as prompt initiation of treatment, in some instances recommended within 20 hours of birth, has been associated with improved outcomes [7].

## Case presentation

**Figure 1:**
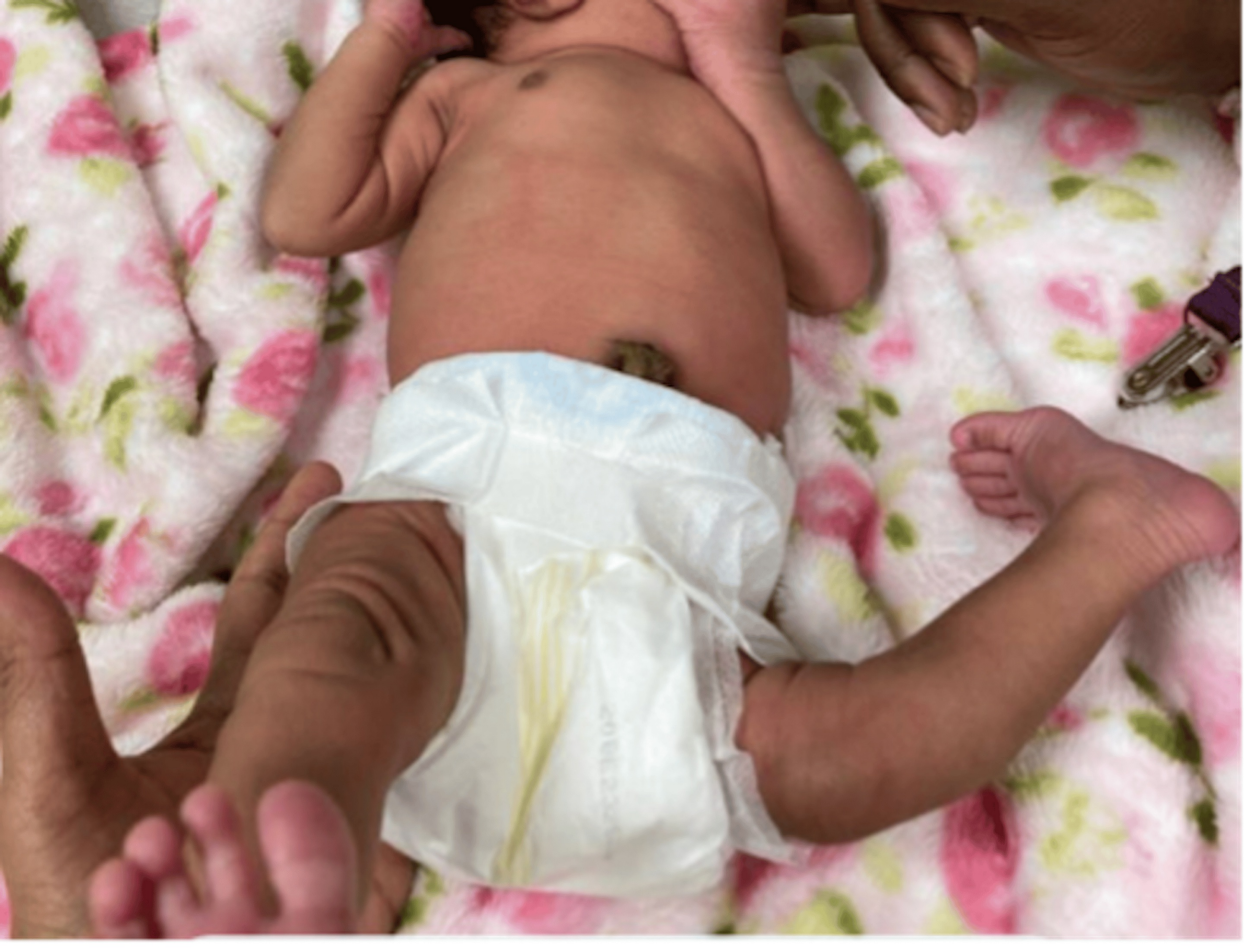
Left knee dislocation with abnormal hyperextension of the left knee at two days of age.

A two-day-old term female presented for her initial newborn visit in the outpatient pediatric clinic with abnormal hyperextension of the left knee noted at birth (Figure 1).

Prenatal care was unremarkable, and maternal history was non-contributory. She was delivered via spontaneous vaginal delivery at 39 weeks’ gestational age in vertex presentation. APGAR scores were reassuring, 9 at both one and five minutes. After delivery, she was feeding well with normal stool and urine output. The remainder of her physical examination was normal.

During her newborn nursery stay, a radiograph of her left knee was obtained, which demonstrated left knee dislocation. She was referred to pediatric orthopedic surgery and evaluated at six days of age. Imaging confirmed dislocation of the left knee, reported as left congenital genu recurvatum. On physical examination, the orthopedist noted restricted left knee flexion, with no flexion beyond 10 degrees. No additional lower extremity abnormalities were identified. The patient had intact neurovasculature and normal skin integrity without erythema or ecchymoses. Barlow and Ortolani tests were negative bilaterally, making DDH less likely.

**Figure 2:**
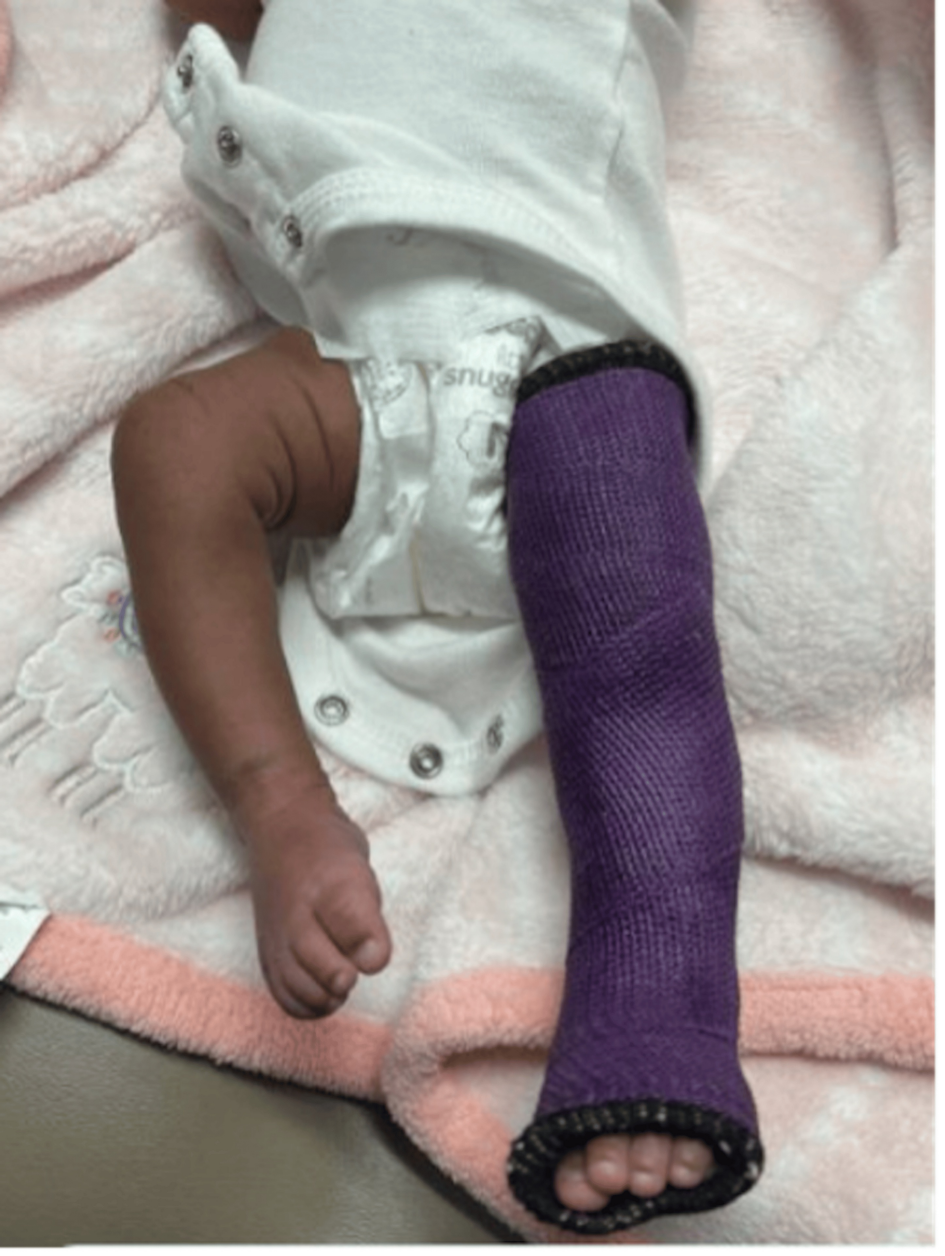
Left lower extremity in long leg cast demonstrating reduced knee joint with 10 degrees of flexion.

She was initially treated with a long straight leg cast in 10 degrees of knee flexion at day six of life (Figure 2). One week later, at 13 days of age, the patient’s left knee flexion improved to greater than 90 degrees.

**Figure 3:**
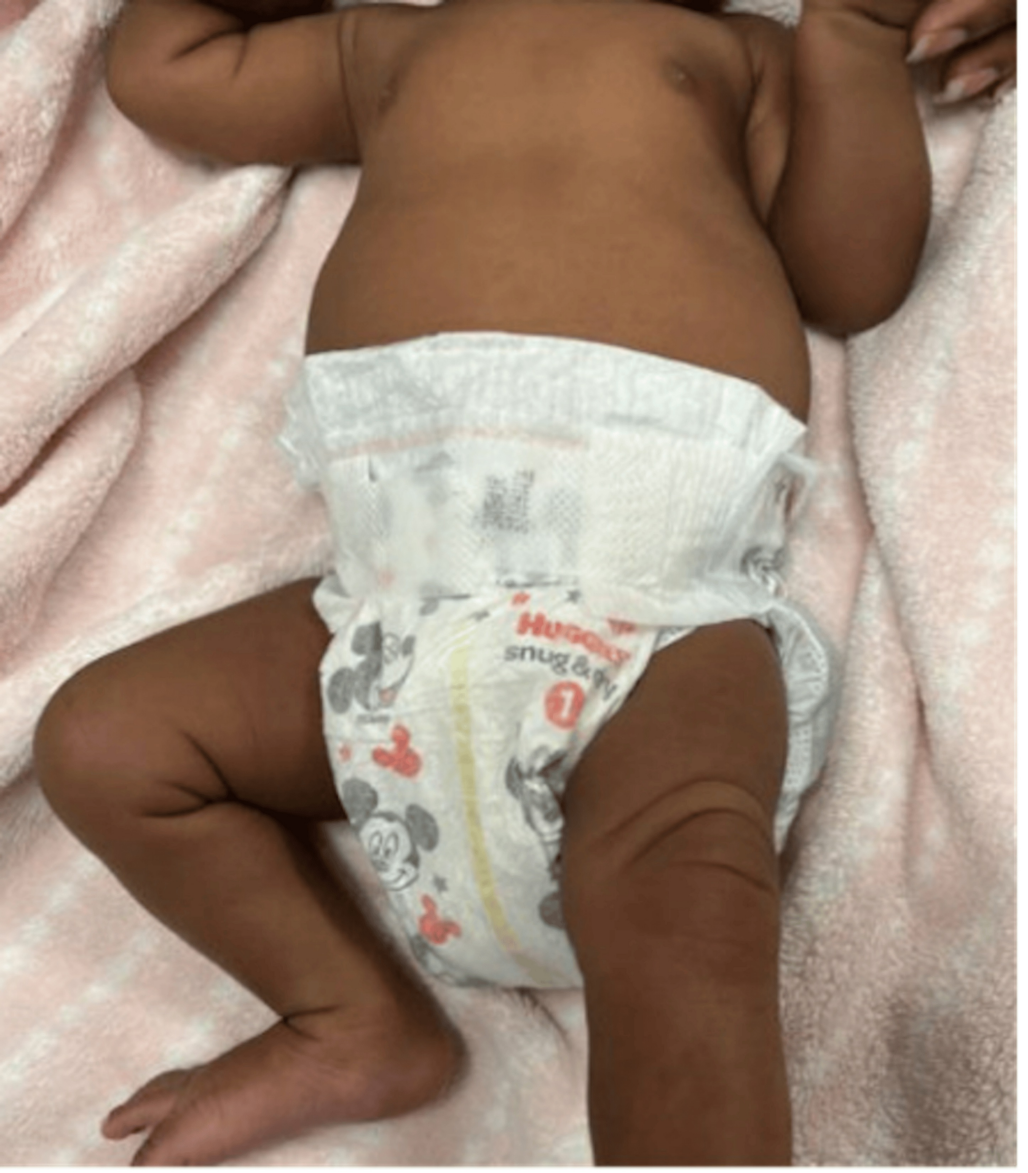
Left lower extremity at three weeks of age with left knee reduced and no longer dislocated.

She was then transitioned to a Pavlik harness, set to hold the knee in 90 degrees of flexion and 60 degrees of abduction, worn 23 hours a day. At her three-week follow-up visit, on day 27 of life, she demonstrated improved range of motion and spontaneous leg movements, with her parents noting kicking of her left leg (Figure 3).

A new Pavlik harness was applied for continued full-time wear. After a total of eight weeks in the Pavlik harness, the infant, now two months old, had full flexion and extension of the left knee with normal alignment. A hip ultrasound was normal, which definitively ruled out DDH. A follow-up radiograph of the left knee and leg, at 12 months of age, demonstrated no dislocation or destructive processes. At 19 months of age, the patient was re-evaluated in the pediatric orthopedic clinic. She demonstrated full range of motion in the left knee and was able to walk and run with a normal gait. A mild five-degree hyperextension of the left knee compared to the right was noted, a finding occasionally seen in patients following casting.

## Discussion

In this case, we present a newborn female with idiopathic left CKD successfully treated with an initial long leg cast for one week, followed by eight weeks in a Pavlik harness to maintain correction. Pavlik harness treatment, while commonly recognized among pediatricians as a treatment for DDH, has also shown utility in CKD. Aside from isolated hyperextension and dislocation of the left knee, the patient had no other health concerns or syndromes associated with CKD, such as Ehlers-Danlos, Larsen, or arthrogryposis [8]. In the setting of these associated syndromes, treatment of CKD can be refractory to conservative management and require operative treatments.

The Pavlik harness is traditionally used for newborns with DDH to maintain proper hip joint alignment, as well as to maintain flexion and positioning in the lower extremities. In the case of CKD, literature indicates that due to the thigh muscles spanning across both the hip and knee joints, the Pavlik harness can leverage this anatomical arrangement and gradually facilitate the spontaneous reduction of the dislocated knee through controlled positioning and immobilization. The Pavlik harness, therefore, is another option to treat cases of congenital knee dislocation in an effective and conservative manner, especially when applied early in reducible or mild cases [6].

The Pavlik harness may offer a benefit of stabilizing the hips in infants who might be at risk for DDH as part of “packaging disorders” noted in cases with reduced intrauterine space, before being able to confirm DDH with a hip ultrasound at six weeks of age. In the event where casting fails or CKD is more severe, surgical interventions, most often quadriceps lengthening, may be required [4]. While outcomes are generally favorable for both non-operative and operative treatments, potential complications include quadriceps insufficiency, ligamentous laxity, valgus deformity following operative management [9], or persistent hyperextension in those managed with casting [4].

## Conclusions

CKD is a rare but clinically significant condition that can present as an isolated finding in otherwise healthy newborns. Early recognition and timely intervention are critical for optimal outcomes. This case highlights the successful use of a conservative management approach, initial casting followed by a Pavlik harness, to achieve anatomical and functional correction without the need for surgery. The Pavlik harness, though more commonly associated with the treatment of DDH, demonstrates utility in selected cases of CKD by leveraging musculoskeletal anatomy to promote gradual joint reduction. Increased awareness among pediatricians of CKD’s presentation and management options may help avoid delays in treatment and reduce the need for invasive procedures. Long-term follow-up remains essential to monitor for residual deformities or functional limitations.

## References

[1] Palco M, Rizzo P, Sanzarello I, Nanni M, Leonetti D (2022). Congenital and bilateral dislocation of the knee: case report and review of literature. Orthop Rev (Pavia).

[2] Drennan JC (1993). Congenital dislocation of the knee and patella. Instr Course Lect.

[3] Morales-Roselló J, Loscalzo G, Hueso-Villanueva M, Buongiorno S, Jakaitė V, Perales-Marín A (2022). Congenital knee dislocation, case report and review of the literature. J Matern Fetal Neonatal Med.

[4] Curtis BH, Fisher RL (1969). Congenital hyperextension with anterior subluxation of the knee. Surgical treatment and long-term observations. J Bone Joint Surg Am.

[5] Shah NR, Limpaphayom N, Dobbs MB (2009). A minimally invasive treatment protocol for the congenital dislocation of the knee. J Pediatr Orthop.

[6] Iwaya T, Sakaguchi R, Tsuyama N (1983). The treatment of congenital dislocation of the knee with the Pavlik harness. Int Orthop.

[7] Ko JY, Shih CH, Wenger DR (1999). Congenital dislocation of the knee. J Pediatr Orthop.

[8] Mottershead NJ, Patel UD, Reynolds P (2012). Congenital dislocation of the knee. Arch Dis Child Fetal Neonatal Ed.

[9] Oetgen ME, Walick KS, Tulchin K, Karol LA, Johnston CE (2010). Functional results after surgical treatment for congenital knee dislocation. J Pediatr Orthop.

